# Efficacy of Divinylbenzenic Resin in Removing Indoxyl Sulfate and P-cresol Sulfate in Hemodialysis Patients: Results from an In Vitro Study and an In Vivo Pilot Trial (xuanro4-Nature 3.2)

**DOI:** 10.3390/toxins12030170

**Published:** 2020-03-10

**Authors:** Maria Teresa Rocchetti, Carmela Cosola, Ighli di Bari, Stefania Magnani, Vanessa Galleggiante, Letizia Scandiffio, Giuseppe Dalfino, Giuseppe Stefano Netti, Mauro Atti, Roberto Corciulo, Loreto Gesualdo

**Affiliations:** 1 Department of Emergency and Organ Transplantation, Nephrology, Dialysis and Transplantation Unit, “AldoMoro” University, 70124 Bari, Italy; melacosola@gmail.com (C.C.); ighli.dibari@uniba.it (I.d.B.); giuseppe.dalfino@gmail.com (G.D.); r.corciulo51@gmail.com (R.C.); 2 Aferetica SRL, 40138 Bologna, Italy; stefania.magnani@aferetica.com (S.M.); vanessa.galleggiante@libero.it (V.G.); letizia.scandiffio@gmail.com (L.S.); mauro.atti@aferetica.com (M.A.); 3 Clinical Pathology Unit and Center for Molecular Medicine, Department of Medical and Surgical Sciences, University of Foggia, 71122 Foggia, Italy; giuseppestefano.netti@unifg.it

**Keywords:** indoxyl sulfate, p-cresyl sulfate, microbiota, synbiotic, sorbents, hemodialysis, chronic kidney disease

## Abstract

High serum levels of microbiota-derived uremic toxins, indoxyl sulfate (IS) and p-cresyl sulfate (PCS), are associated with chronic kidney disease (CKD) progression and cardiovascular complications. IS and PCS cannot be efficiently removed by conventional hemodialysis (HD), due to their high binding affinity for albumin. This study evaluates the efficacy of a divinylbenzene-polyvinylpyrrolidone (DVB-PVP) cartridge and a synbiotic to reduce uremic toxins in HD patients. First, the in vitro efficacy of DVB-PVP in adsorbing IS and PCS was evaluated. Second, a randomized, placebo-controlled pilot study in HD patients was carried out to establish whether the administration of a synbiotic, either individually and in association with DVB-PVP-HD, could reduce the production of uremic toxins. In vitro data showed that DVB-PVP resin removed a mean of 56% PCS and around 54% IS, after 6 h of perfusion. While, in the in vivo study, the DVB-PVP cartridge showed its adsorbing efficacy only for IS plasma levels. The combination of synbiotic treatment with DVB-PVP HD decreased IS and PCS both at pre- and post-dialysis levels. In conclusion, this study provides the first line of evidence on the synergistic action of gut microbiota modulation and an innovative absorption-based approach in HD patients, aimed at reducing plasma levels of IS and PCS.

## 1. Introduction

Protein-bound uremic toxins (PBUTs), such as indoxyl sulfate (IS) and p-cresyl sulfate (PCS), accumulate in chronic kidney disease (CKD), especially in patients with end-stage renal disease (ESRD) [1,2,3]. IS and PCS, derived from the gut microbiota metabolism of dietary amino acids, tryptophan, and tyrosine, are absorbed by the gastrointestinal tract and sulfated in the liver, then circulate in blood non-covalently and competitively bind to albumin and are actively excreted in urine via the basolateral organic anion transporters in the renal tubules [4]. The circulating levels of IS and PCS increase with the progression of renal failure in CKD, reaching their maximal concentration in patients undergoing hemodialysis (HD) treatment, where higher levels predict poor clinical outcomes [5,6] even in elderly HD patients [7]. These microbial-derived metabolites are emerging as non-traditional cardiovascular risk factors in CKD [8], as evidenced by meta-analyses and cohort studies that associate them to all-cause or cardiovascular mortality [7,9,10]. Compared to the classical uremic markers, such as urea and creatinine, which are far more efficiently removed by dialysis treatment [11], IS and PCS are not sufficiently removed because of their high binding affinity to albumin, which makes only a low (2–5%) [1] free fraction available to diffusion in the dialysate. Therefore, PBUTs represent a challenge for extracorporeal renal replacement strategies because only the unbound solute can pass through conventional dialysis membranes. Despite the progress in dialysis treatment, the reduction rate (RR) of total IS (protein-bound and unbound IS) with standard HD remains lower than 35% [1,11,12], unless longer dialysis sessions, raising the dialysate flow and dialyzer size [13,14,15], or advanced synthetic high-flux membranes [16] are used for extracorporeal renal replacement therapy. In these cases, the RRs of total IS hardly reach values of 50% (40% for total PCS), which is still not enough as its plasma levels often remain highly elevated after HD [13,16,17]. Moreover, IS and PCS circulating levels rapidly rise again before the following dialysis session [18]; therefore, a therapeutic strategy, synergistically acting both upstream, reducing the production of uremic toxins, and downstream, increasing their removal, is expected to be advantageous. 

In recent years, the use of sorbents in hemoperfusion (HP) or plasma perfusion has become common, mostly in combination with other blood purification techniques [19]. Adsorption is considered a complementary mechanism for solute removal, due to the intrinsic capability of sorbents to bind molecules based on chemical affinity rather than membrane permeability, sieving coefficient, and the molecular weight cut-off, typical mechanisms influencing diffusion and convection. In HP, blood circulates through a cartridge containing the solid sorbent material. In clinical practice, several new sorbents have been proposed to specifically remove target molecules, including PBUTs [20,21]. The in vivo efficacy of sorbents to remove the uremic toxins predicted by in vitro studies is still debated [21]. In this field, the most promising adsorbent materials are based on divinylbenzene coated with polyvinylpyrrolidone (DVB-PVP) or cellulose with hexadecyl chains for their high hydrophobic nature and biocompatibility, making them appropriate for the direct use in whole blood. These materials have been reported to remove molecules with chemical characteristics similar to PBUTs, such as albumin-bound bilirubin [22,23] or hydrophobic cytokines [24,25], in several clinical settings. These compounds have a hydrophobic characteristic similar to the microbial-derived metabolites, justifying the rationale for the use of DVB-PVP or cellulosic resins for the absorption of either IS and PCS.

Gut microbiota proteolytic dysbiosis is present in CKD and especially in HD patients, and contributes to an increased generation of PBUTs that progressively accumulate as the renal excretory capacity declines [26]. Reducing PBUTs is a priority, given their inflammatory, pro-oxidant, and profibrotic effects, and their role in the increased cardiovascular risk in CKD patients [8].

Acting on PBUTs production at the colonic level is a strategy that has already been experimented with by other studies. Indeed, some trials carried out with HD patients attempted to lower the production rates of microbial-derived PBUTs, acting on microbiota modulation through probiotic [27,28,29], prebiotics [30,31], and synbiotics [32], with contrasting results. 

These premises represent the rationale for the NATURE study, in which we aimed to use a double-edged approach to the reduction of PBUTs in HD patients, consisting in the upstream reduction in their production at colonic level, and in the downstream improvement in the efficacy of their removal by the dialysis treatment.

The present study was designed first, to evaluate the in vitro efficacy of two sorbent resins with different hydrophobic nature, divinylbenzene (DVB-PVP) and cellulose, in adsorbing IS and PCS. Second, a pilot clinical study in HD patients was carried on to establish whether: i) the administration of an innovative synbiotic formulation could reduce the production of PBUTs; ii) the innovative HD session using DVB-PVP-cartridges could remove IS and PCS more efficiently than standard bicarbonate dialysis, and iii) their association could act in a synergistic fashion in removing PBUTs.

## 2. Results

### 2.1. Removal of IS and PCS by DVB-PVP and Cellulose Resins: In Vitro Study

**Figure 1:**
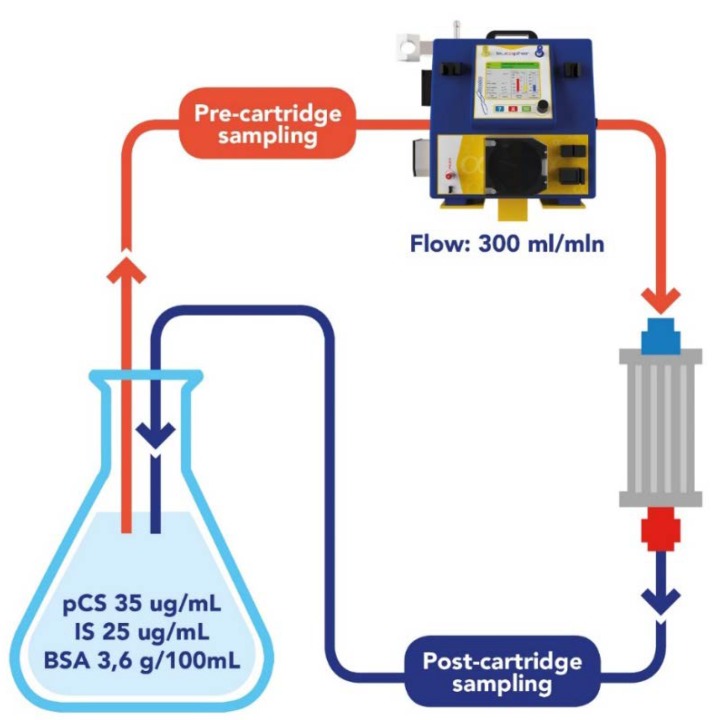
In vitro experiment. Recirculation of an experimental solution in a closed circuit containing the resin sorbent to be tested. Solution samples to be tested were collected after passing through the resins after 1, 3, and 6 hours.

**Figure 2:**
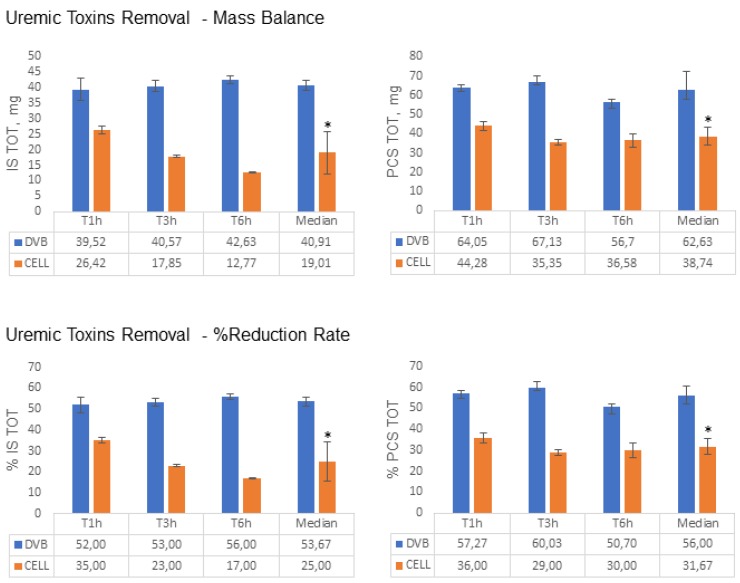
Amount of total IS and PCS removed from the experimental solution after passing through the cartridge containing the DVB resin and cellulose, respectively, at different time points. Data are expressed as median ± SD for Mass Balance = (Cfinal-Cinitial) * Total Volume and for RR: Removal Rate = (Cinitial − Cfinal)/Cinitial * 100.

At first, resin adsorption capacity towards IS and PCS was assessed in vitro (Figure 1). DVB-PVP resin adsorbed total IS and PCS to a greater extent than cellulose resin. Indeed, after 6 hours of perfusion, DVB-PVP removed a mean of 40.9 ± 1.6 mg of total IS compared to cellulose (19.0 ± 6.8 mg; *p* = 0.04) and a mean of 62.6 ± 9.8 mg of total PCS compared to cellulose (38.7 ± 4.8 mg; *p* = 0.04) starting from a total amount of 75.4 mg of IS and 115.7 mg of PCS in the solution (Figure 2 and Table 1). 

Likewise, free portions of IS and PCS were also more efficiently absorbed by DVB-PVP resin compared to cellulose. Indeed, after 6 hours perfusion, DVB-PVP removed a mean of 8.17 ± 0.48 mg of free IS and 10.41 ± 1.30 mg of free PCS compared to cellulose (1.79 ± 0.55 mg of free IS and 2.30 ± 0.71 mg of free PCS, respectively; *p* = 0.04 for both. Table 1). In terms of percentage of the IS and PCS initial amounts removed from the experimental solution, DVB-PVP resin adsorbed, after 6 hours of perfusion, a mean of 53.7 ± 2.0% of 0.1mM IS, and a mean of 56.0 ± 4.7% of 0.177 mM PCS compared to cellulose resin (25.0 ± 9.0% total IS and 31.7 ± 3.7% total PCS, respectively; *p* = 0.04 for both) (Figure 2, Table 1). Considering the free fractions, DVB-PVP resin reduced free IS of 66.0% ± 4.0% (mean) and free PCS of 70.0 ± 8.5% (mean) compared to 15.3 ± 4.9% (mean) reduction in both free IS and PCS by cellulose. 

In addition, we evaluated the effect of the resins on the variation of IS and PCS albumin binding, and we found that the percentage of IS and PCS albumin binding (%PB) increased during the six hours of perfusion through DVB-PVP, while decreased during six hours of perfusion through cellulose, corresponding to the pH values. Data are reported in Supplementary Materials (Table S1: %PB of IS and PCS, and pH values registered in the experimental solution at start condition and at the end of the considered time points).

### 2.2. Clinical Trial

**Figure 3:**
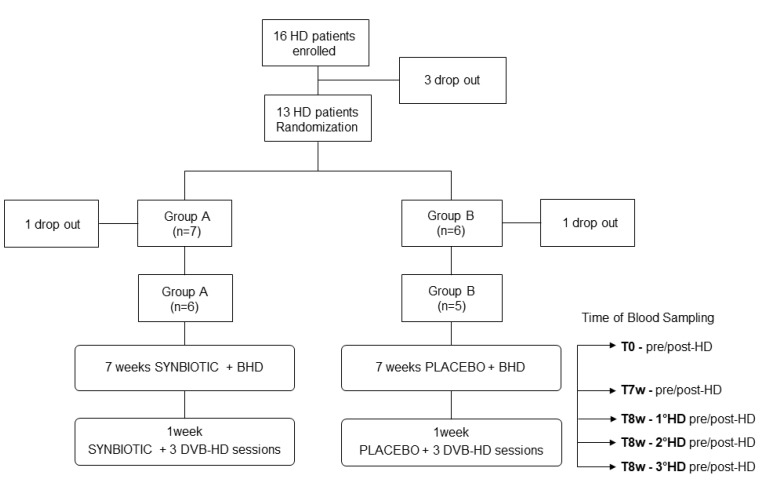
Study Design.

Table 2 shows the characteristics of 11 out of 16 hemodialysis patients who completed this pilot study, at baseline, after 7 weeks of the synbiotic or placebo treatment following the traditional bicarbonate hemodialysis (BHD) and after one week of synbiotic or placebo treatment combined with three sessions of DVB-PVP cartridge hemodialysis (DVB-PVP HD) (Figure 3). Each patient underwent routine blood examinations at baseline (T0), at the end of the 7-weeks synbiotic or placebo treatment (T7w), and at the end of the eighth week (T8w). IS and PCS plasma levels were measured pre- and post-HD at each time point, as reported in Figure 3. 

DVB-PVP HD treatment reduced the albumin plasma levels by 0.4 g/dL compared to BHD in the synbiotic-treated patient group, while it reduced the albumin plasma levels by 0.3 and 0.4 g/dL as compared to BHD and baseline condition in control group (placebo) (Table 2).

### 2.3. Effects of Synbiotic on IS and PCS Plasma Levels 

**Figure 4:**
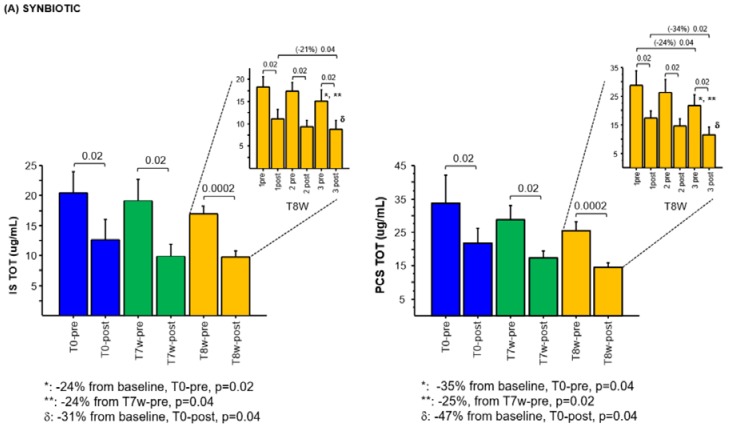

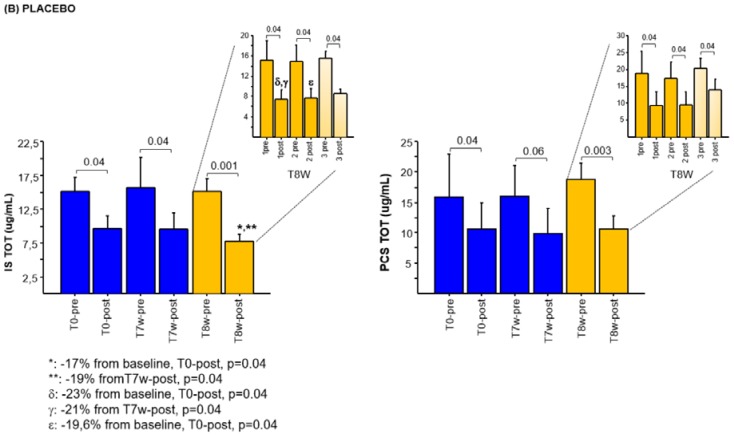
Plasma concentrations of the total IS and PCS in the two groups (**A** Synbiotic and **B** Placebo) of dialysis patients: (**A**) at baseline (T0-pre/post-dialysis); after 7 weeks of synbiotic treatment (T7w-pre/post-dialysis); mean values before and after the three dialysis sessions with the DVB-cartridge with the synbiotic treatment at week 8 (T8w-pre/post-dialysis). At the top right of each chart, the values before and after each dialytic session carried out at week 8 are reported: first (1-pre/post), second (2- pre/post), and third (3-pre/post) dialysis session. (**B**) at baseline (T0-pre/post-dialysis); after 7 weeks of placebo treatment (T7w-pre/post-dialysis); mean values before and after the three dialysis sessions with DVB-cartridge with the placebo treatment at week 8 (T8w-pre/post-dialysis). At the top right of each chart, the values before and after each dialytic session carried out at week 8 are reported: first (1-pre/post), second (2-pre/post), and third (3-pre/post) dialysis session. Data are expressed as mean ± SE. (Wilcoxon test).

There was no statistically significant difference, at baseline, between the synbiotic (A) and placebo (B) group for both plasma IS (*p* = 0.36) and PCS levels (*p* = 0.25). Although not statistically significant, the seven-weeks synbiotic treatment showed a reduction trend in pre- and post-dialysis levels (μg/mL) of total IS (19.9 ± 7, T7w-pre; 9.9 ± 5, T7w-post) and PCS (28.9 ± 9, T7w-pre; 17.2 ± 5, T7w-post) compared to baseline (20.5 ± 8, T0-pre and 12,6 ± 8, T0-post for IS; 33.7 ± 18, T0-pre and 21.7 ± 9, T0-post for PCS) (Figure 4A), while in the control group the placebo administration did not (Figure 4B). 

### 2.4. Effects of the Combined Synbiotic with Innovative Dialysis Treatment on IS and PCS Plasma Levels

The combination of synbiotic treatment with DVB-PVP HD in group A, progressively, in the course of the three dialysis sessions, reduced the production and increased the removal of total IS and PCS as compared to the placebo group (yellow columns of Figure 4A,B). Indeed, total pre-dialysis levels of IS decreased 24% (*p* = 0.02; *p* = 0.04) in the synbiotic group of patients, from the baseline (20.5 ± 8, T0-pre) and the end of the 7^th^ week (19.9 ± 6, T7w-pre) to the third dialysis session of the 8^th^ week (15.1 ± 6, T8W-3pre). Total pre-dialysis levels of PCS decreased 35% (*p* = 0.04) and 25% (*p* = 0.02) in the synbiotic group of patients, from the baseline (33.7 ± 18, T0-pre) and from the end of the 7^th^ week (28.9 ± 9, T7w-pre) to the third dialysis session of the 8^th^ week (21.7 ± 9, T8W-3pre) (Figure 4A). Likewise, post-dialysis total IS plasma levels decreased 31% (*p* = 0.04) from baseline (12.6 ± 8, T0-post) to the third dialysis session of the 8th week (8.73 ± 5, T8W-3post) and of 21% (*p* = 0.04) from the first (11.0 ± 5.1, T8W-1post) to the third DVB-PVP HD session of the 8^th^ week (8.7 ± 5, T8W-3post) (Figure 4A). In the same way, post-dialysis total PCS plasma levels decreased 47% (*p* = 0.04) from baseline (21.7 ± 9, T0-post) to the third dialytic session of the 8^th^ week (11.4 ± 7, T8W-3post) and of 34% (*p* = 0.02) from the first (17.4 ± 6, T8W-1post) to the third DVB-PVP HD session of the 8^th^ week (11.4 ± 6, T8W-3post) (Figure 4A).

### 2.5. Effects of the Innovative Dialysis Treatment on IS and PCS Plasma Levels

In the control group (B), the pre-dialysis reduction in total IS and PCS plasma levels was not observed during the placebo administration (Figure 4B). Conversely, DVB-PVP HD treatment showed a trend of post-dialysis reduction in IS and PCS greater than BHD, reaching a statistical significance only for IS (Figure 4B). In detail, after the first DVB-PVP HD session, post-dialysis IS plasma levels (7.4 ± 4, T8W-1post), reduced to 23% (*p* = 0.04) and 21% (*p* = 0.04) compared to baseline (9.6 ± 4, T0-post) and to T7w-post (9.4 ± 5), respectively (Figure 4B). The second DVB-PVP HD session significantly reduced post-HD IS plasma levels (7.7 ± 4, T8W-2 post) by 19.6% (*p* = 0.04) compared to baseline (9.6 ± 4, T0-post). Two patients of this group did not complete the third DVB-PVP HD session owing to the lowering of blood pressure during the treatment, not allowing the observation of the trend towards reduction in IS and PCS levels after the third experimental session (last column, Figure 4B) (last columns in light yellow, Figure 4B). 

Likewise, free IS and PCS plasma levels followed the same trend of reduction as the total IS and PCS, in all pre-dialysis plasma samples of HD patients treated with symbiotic (group A). Free IS and PCS values and the reduction ratios (RRs) for total and free IS and PCS are reported in Supplementary Materials (Figure S1: Plasma concentrations of the free IS and PCS in the two groups (A Synbiotic and B Placebo) of HD patients. Table S2: Reduction ratio for total and free IS and PCS concentration at the different dialysis sessions during the trial in the synbiotic-treated group (group A) and placebo-treated group (group B)). 

In accordance with in vitro data, we observed increased IS and PCS protein binding (%PB) in post-dialysis samples along with an increase in pH during the DVB-PVP HD treatment. Details are provided in Supplementary Materials (Table S3: Pre- and post- %PB of indoxyl sulfate and p-cresyl sulfate, and pH values registered in the plasma of HD patients undergoing DVB-PVP HD beside synbiotic or placebo treatment).

## 3. Discussion

This study was performed to compare the efficacy of two different adsorbent resins to reduce IS and PCS by an in vitro study, and to test the efficacy of IS and PCS removal by the DVB-PVP cartridge in vivo, by a small-scale randomized controlled trial in HD patients. Furthermore, the efficacy of the synbiotic NATUREN G, singularly for 7 weeks, and in combination with the innovative DVB-PVP HD, for 1 week, was tested. The aim of this strategy was to reduce PBUTs production by gut microbiota modulation and to increase their removal by improving blood purification therapy in patients undergoing HD, for their better survival [5,7,9] and quality of life. Sorbents, routinely used for many years in drug intoxication [33] and poisoning, have been more recently employed in other clinical application, such as sepsis [34,35], acute kidney injury [36], and, lately, to remove molecules not easily cleared by HD, such as PBUTs [20,21,37,38,39]. In one of the first attempts to assess the capacity of the sorbent system to increase protein-bound solutes’ removal by HD, it was demonstrated how the addition of activated charcoal to the dialysate could increase the clearance of indican, p-cresol, and PCS. In these in vitro dialysis experiments, for each protein-bound solutes, the clearance with charcoal added to the dialysate, doubled the clearance without charcoal, while it had no effect on the clearance of unbound solutes, such as urea [39]. 

Our in vitro data showed that DVB-PVP resin was more effective in adsorbing IS and PCS than cellulose, removing a mean of 56% PCS and around 54% IS, after 6 h of perfusion, from an experimental solution containing mean IS and PCS concentrations comparable to those present in hemodialysis patients [1]. In the in vivo study, the DVB-PVP cartridge showed its adsorbing efficacy only for IS plasma levels if compared to the baseline, as evidenced in the placebo group, after the first two DVB-HD sessions (Figure 4B). Two of the five patients in the placebo group did not complete the third DVB-PVP HD session owing to the lowering of blood pressure during the treatment, not allowing the observation of the trend towards a reduction in IS and PCS levels also after the third experimental session. We are unable, actually, to explain the discrepancy between in vitro and in vivo data on PCS removal. We can only assume that the in vivo relationship between PCS and resin is affected by a variety of conditions hard to reproduce in an in vitro setting, where standardized conditions are an effort to eliminate every kind of interference that might influence the ability by the resins to remove IS and PCS and to characterize the resins’ behavior. Differently, many factors could affect the IS and PCS concentration in vivo, such as their binding with proteins other than albumin. We are aware that longer follow up and more cases would provide a better assessment of PCS removal. 

Similarly, a recent study reported that hexadecyl-immobilized cellulose beads (HICB) adsorbed IS free (55.4% of 1 mM IS solution) in vitro, much more than polysulfone dialysis membrane and polymethyl methacrylate membrane, but the clinical trial to use a (HICB)-containing column did not show an effect in reducing serum PBUTs levels in HD patients [21]. Sandeman and coworkers [37] tested the in vitro efficacy of IS and PCS adsorption by nanoporous activated carbon monolith prototype finding a reduction of IS (−85%) and PCS (−78%) concentration after 60 minutes perfusion through it. Furthermore, they showed that nanoporous monoliths were able to remove IS (−83%) and PCS (−75%) after 30 min of continuous perfusion of 20 mL of the post high flux HD patient blood samples, observing, in addition, an albumin drop of 23%. In the final conclusions, the authors recognized that further work is necessary to develop and assess a scaled-up device that can be used in conjunction with a dialysis circuit [37]. Finally, in a recent in vitro study, the removal of a broad range of uremic toxins, including IS and hyppuric acid (HA), from human plasma by a carbon-based nanoporous sorbent material (CMK-3 type) which has two distinct pore domains, micropores, and mesopores, was investigated [38]. The authors found that the amount of IS and HA/m2 adsorbed by CMK-3 was slightly higher than carbon-based sorbents with predominant mesoporosity and much higher than carbon-based sorbents with predominant microporosity. The studied materials did not show a tendency to lower total plasma protein levels through 4 hours of direct plasma–sorbent contact showing high potential to be used in extracorporeal blood purification treatments [38].

In our opinion, the interesting data emerging from this pilot study are represented by the combined effect of the synbiotic and the DVB-PVP HD on the reduction in IS and PCS plasma levels in HD patients. In this trial, the use of NATUREN G alone apparently did not seem to be sufficient in reducing pre-HD plasma levels of IS and PCS in traditional bicarbonate dialysis, after 7 weeks of treatment, as compared to baseline and placebo group. 

Indeed, failure to decrease the pre-dialysis plasma levels of IS and PCS after synbiotic treatment may be due to the multi-compartmental distribution of IS and PCS, as suggested by the two-compartment kinetic model for protein-bound solutes [40]. In this model, all the solutes showed a substantially slower decline of concentration in the extraplasmatic compartment, which is the place where they mainly exert toxicity. In this context, the synbiotic may have gradually reduced the production of IS and PCS over the 7 weeks of treatment, progressively depleting the deposits of uremic toxins in the extraplasmatic compartment [40], and leaving their plasma levels unchanged. The decrease in plasma levels of IS and PCS became visible from the eighth week. At the same time, the innovative DVB-PVP dialysis, adsorbing higher percentages of IS and PCS, made possible the further reduction in uremic toxins both at pre- and post-dialysis levels, resulting in a decrease in the pre-dialysis IS and PCS by 24% and 35%, respectively, and of the post-dialysis IS and PCS by 31% and 47%, respectively, compared to the baseline. This synergy was not found in the placebo group, where it was possible to evaluate the isolate better ability of DVB-PVP HD to remove IS compared to BHD. Effectively, a trend of increased RR% of total IS and, to a lesser extent for total PCS, in both groups A and B, after each DVB-PVP HD session was observed (Table S2: Reduction ratio for total and free IS and PCS concentration at the different dialysis session during the trial in the synbiotic-treated group (group A) and placebo-treated group (group B)), which was more evident in the placebo group, suggesting a better capacity of DVB-PVP HD to remove IS and PCS as compared to BHD. 

Attempts to manipulate PBUTs production by acting on gut microbiota have been made in the last years. The rationale of this approach is justified by the plastic capacity of gut microbiota to respond to environmental stimuli, primarily nutrition and supplementation [26]. In this regard, our group recently demonstrated that dietary schemes, such as Mediterranean diet and a very low protein diet, are able to modify, in a saccharolytic fashion, gut microbiota in CKD patients and to reduce PCS and IS [41]. 

Analogously, supplementation with substances capable of inducing beneficial bacteria colonization and/or selective growth, namely probiotics, prebiotics, and synbiotics, seems to represent a promising strategy to reduce PBUTs production [26]. 

Few papers studied the effects of probiotic administration in HD patients with discrepant results. One trial evidenced the differential efficacy in IS reduction by *Bifidobacterium* supplements, where the formulation (gastro-resistant capsule vs. powder) seems to be a key element in probiotic colonic delivery [27]. Very recently, the group of Eidi demonstrated the efficacy of a *Lactobacillus Rhamnosus* capsule formulation to reduce phenol and PCS in an RCT carried out in HD patients [28]. On the contrary, the probiotic supplementation of the *Streptococcus thermophilus*, *Lactobacillus acidophilus* e *Bifidobacterium longum* capsule failed to reduce uremic toxins and inflammatory markers in HD patients [29]. In peritoneal dialysis patients, a *Bifidobacterium* spp + *Lactobacillus* formulation seems to decrease inflammatory cytokine levels, but the trial did not analyze reductions in PBUTs levels [42]. Other trials tested the action of prebiotic supplementation in HD patients. Meijers and coworkers demonstrated the efficacy of a 4-weeks’ supplementation of oligofructose-enriched inulin in reducing both PCS circulating levels and production rates in HD patients, while IS levels remained unchanged [30]. Differently, the group of Esgalhado recently evidenced the effects of resistant starch in reducing IS and IL-6 [31]. The synbiotic association of prebiotics and probiotics has also been tested in HD. The group of Nakabayashi reported a reduction in PCS and amelioration in stool transit and consistency after 2 weeks of a *Lactobacillus casei Shirota*, *Bifidobacterium breve Yakult*, and galactooligosaccharides formulation [32]. Other trials carried out with synbiotic formulations in HD patients did not investigate the modulation of PBUTs levels, rather revealing other beneficial effects on gut microbial composition [43] and amelioration of biomarkers of glucose homeostasis, inflammation, and oxidative stress [44].

The lack of an observed effect in our population by the synbiotic administration after seven weeks could be attributed to several reasons. First of all, the small sample size prevented us from observing even small changes in PBUTs level. Second, a different, gastro-resistant capsule formulation instead of the powder one could have guaranteed better gut colonization. Third, the HD population is characterized by an advanced uremic status in comparison to CKD patients at intermediate stages. Evidently, an isolate synbiotic supplementation is not enough to counterbalance the dysbiotic state continuously fostered by the urea colonic influx. A harder strategy is likely necessary, and the results we observed during the overlapping period between the synbiotic supplementation and the DVB-PVP experimental treatment seem to support this interpretation and justify the experimentation of this combined approach for longer periods or for repeated cycles. 

Adsorption relies on the direct binding of solutes to sorbent materials contained within a cartridge, which depends on the sorbent–solute chemical affinity. One of the main drawbacks of extracorporeal blood purification techniques (i.e., cut-off membranes, high-volume hemofiltration) is the loss of nutrients, such as albumin, useful bioactive molecules, and drugs, therefore, minimizing unwanted molecules loss is an important challenge for sorbent materials research [45]. Indeed, the two resins under investigation also differed in albumin adsorption, with greater adsorption by the DVB-PVP resin. Although the maximum loss with DVB-PVP resin in vitro was about 14%, the in vivo albumin-leaking, due to the use of three consecutive HD sessions with the DVB-PVP cartridge, in both synbiotic/placebo-treated groups, was around 8%, an accepted albumin loss, which could be considered remaining in the normal range concentration [37].

The DVB-PVP cartridge here investigated was able to modulate hydrophobic molecules up to a molecular weight of 60 kDa, which are involved in various critical pathologies, and this ability is widely observed in the literature [24,25,46]. Its capability to remove albumin (65–70 kDa), as well as other adsorbents and other proteins from the blood, has not been properly investigated yet. In the literature, Schädler and coworkers [47] showed a limited albumin loss from baseline after the treatments, indicating that the device had no significant impact on these values. Moreover, Gemelli and coworkers [23] specifically studied the behavior of the investigated adsorbent material regarding protein-bound hepatic toxins, such as bilirubin, observing the ability to irreversibly adsorb bilirubin without affecting albumin, which remained stable (changes less than 5%). Apart from these experiences, we are not aware of any other study focused on albumin behavior with the DVB-PVP sorbent or any comparative studies among different extracorporeal techniques. Certainly, this should be investigated in future studies. Therefore, we wondered whether the removal of IS and PCS by DVB-PVP HD treatment was linked to the loss of albumin. Based on the observed data, we cannot exclude that the reduction in PBUTs is also due to the loss of albumin, although all the statistically significant reductions in IS and PCS plasma levels in post-HD samples of synbiotic/placebo DVB-PVP HD treated groups were greater than 8%. However, considering the DVB-PVP resin adsorption efficacy recorded in the in vitro study, and given the study limitations, small sample size and only three DVB-PVP HD sessions, we are aware that longer observation periods with a major number of patients are necessary to better investigate the impact of a DVB-PVP sorbent on PBUTs removal in HD patients. 

The increase in %PB for both IS and PCS after dialysis treatment confirmed literature data [48].

However, Deltombe and coworkers cannot explain the changes in %PB during hemodialysis by changes in pH. They hypothesized that the increased percentage of toxins PB during HD could be attributed to the slow restoration of the balance between their protein-bound and the free forms during the dialysis session once the pre-dialysis free fraction has been removed. They confirmed their hypothesis, calculating the difference in reduction ratio (RR) between the free and total concentration of highly-bound IS and PCS, which might imply that equilibrium could not be restored during the dialysis session. Indeed, we found that most of the differences between RRs of the total and free IS and PCS concentrations were statistically significant (Table S2), with the RRs of the free form much higher, which means that only the free fraction can be removed from dialysis and will cause an unbalance with the bound fraction, which is released slowly. Although we found a positive correlation between %PB of IS and PCS and pH, both in vitro and in vivo with DVB-PVP resin, we could just suggest a probable influence of pH in restoring the balance of the free and protein-bound form of uremic toxins in HD patients, being aware of the need for further experiments for confirmation. This information might be useful in the improvement in the PBUTs removal strategies and in the development of innovative ones aiming at the optimization of dialysis.

In conclusion, this study demonstrates DVB-PVP resin efficacy in removing IS and PCS in vitro. Moreover, it represents a proof of concept of the potential validity of a strategy that combines the gut microbiota modulation and an innovative absorption-based approach in HD patients, aimed at reducing plasma levels of IS and PCS. Extended treatment cycles with a larger number of patients are needed to confirm the efficacy and the applicability of the combined strategy synbiotic/DVB-PVP dialysis in uremic patients, with clinical outcomes that need to be studied.

## 4. Materials and Methods

### 4.1. Materials

The solution for all the in vitro experiments was prepared by diluting a stock solution (10X) composed of 1.5 L of phosphate-buffered saline (PBS) 10 mM pH 7.4 (Lonza, Walkersville, MD, USA), 262.95 g of NaCl (Sigma–Aldrich, Saint Louis, MO, USA), 540.54 g of bovine serum albumine (BSA) (Sigma–Aldrich) under slow agitation until complete dissolution at room temperature in a dark condition. At least one hour before the use, 140 mg of PCS potassium salt (synthetized by Organic Chemistry Laboratory of Dipartimento di Farmacia-Scienze del Farmaco, Università di Bari "A. Moro", Consorzio C.I.N.M.P.I.S.), 105 mg of IS potassium salt (Sigma–Aldrich) and 10 mM PBS were added to 350 mL of stock solution under slow agitation, at room temperature in a dark condition to have the final solution (3.5 L) reported in the In vitro study paragraph. Reagents for LC-MSMS analysis were all of mass spectrometry grade: ammonium acetate, methanol, acetonitrile, and distilled water of high–performance liquid chromatography (Ultra CHROMASOLV) grade were purchased from Sigma–Aldrich. Standard PCS ammonium salt was from ALSACHIM (Bioparc, Illkirch, France), and indoxyl-4,5,6,7-d4 sulfate potassium salt was from Toronto Research Chemicals (North York, ON, Canada). 

### 4.2. Cellulose and DVB-PVP Resins

The innovative DVB-PVP cartridge, divinylbenzene coated with polyvinylpyrrolidone (Aferetica, Bologna, Italy), was used for the execution of these experiments. The cartridge was characterized by biocompatible porous polymer beads (diameter of the beads: 450 μm, diameter of the pores: 0.8–5 nm) able to remove substances from whole blood based on pore capture and surface adsorption. The beads determined an estimated total adsorption surface of more than 40.000 m^2^, decreasing high toxins levels efficiently and remaining effective over an extended period (up to 24 hours). Regarding the cellulosic material, a resin made by cellulose functionalized with hexadecyl chains, (Aferetica, Bologna, Italy) was used considering its removal abilities regarding hydrophobic compounds with a molecular weight less than 30 kD, especially β2-microglobulin, without compromising albumin. 

Both these cartridges were chosen in accordance with their technical characteristics of acting on whole blood during hemodialysis procedures thanks to their high hemocompatibility. This is a point worth knowing, considering the technical application of the sorbents according to the clinical trial protocol. 

### 4.3. In Vitro Adsorption Assay

We designed an in vitro study with an experimental solution reproducing in vitro the uremic pathological condition, containing mean concentrations of indoxyl sulfate (IS) and p-cresyl sulfate (PCS) analogous to those present in hemodialysis patients, to compare the efficacy of two sorbents, namely cellulose and DVB-PVP, to remove uremic toxins. A closed-circuit was used in the experiment, where 3.5 L of 10 mM PBS solution (pH 7.4) containing IS potassium salt (105 mg, 0.12 mM), PCS potassium salt (140 mg, 0.177 mM), NaCl (0.30 M) and bovine serum albumin (BSA, 3.6 g/dL) was pumped by means of a peristaltic pump at 300 mL/min flux (Figure 1). Before the in vitro study, we tested the percentage of IS and PCS protein binding (%PB) to albumin in the experimental solution by quantitative LC-MS/MS analysis, using indoxyl-4,5,6,7-d4 sulfate as internal standard, to evaluate their total and free concentrations. After the LC-MS/MS analysis, the IS recovery was 85%, and PCS recovery was 100%. Measuring total and free IS and PCS of the experimental solution, we calculated 84.15% PB for IS and 87.15% for PCS, which is not the naturally occurred protein-binding percentage (>95%), but which we considered suitable for the study purpose. Adsorption was calculated from the IS and PCS concentration remaining in the perfusing solution after passing through the resin sorbents after 1, 3, and 6 hours. IS and PCS concentration was measured by LC-MS/MS, as described below. BSA concentration was measured by a colorimetric assay based on the Bradford dye-binding method (BioRad Protein assay). Mass balance (MB), removal rate (RR) and percentage protein binding (%PB) of IS and PCS were calculated for both resin sorbents: MB and RR were calculated from the measured initial and final concentration (C) as MB = (Cfinal-Cinitial) × Total Volume; RR = (Cfinal-Cinitial)/Cinitial × 100; %PB was calculated from the measured total (CT) and free (CF) concentrations as %PB = [1 − (CF/CT)] × 100%. 

### 4.4. Clinical Trial: Patients and Study Design

A randomized, placebo-controlled, pilot study in HD patients was set up to verify the hypothesis that an innovative combined approach with the use of a synbiotic and an innovative dialysis cartridge would be effective in reducing circulating levels of microbial uremic toxins and inflammation. 

The study was carried out in accordance with the Helsinki Declaration (IV Adaptation) and the European Guidelines for Good Clinical Practice. The protocol of the study was approved by the Ethical Committee of the Azienda Ospedaliero-Universitaria Consorziale Policlinico of Bari, Italy (Authorization nr. 0642; 17 May 2017). Written informed consent was obtained from all patients. The authors confirm that all ongoing and related trials for this intervention are registered in the ClinicalTrials.gov registry database; because of administrative issues that led to a delay in the ClinicalTrials.gov registration, we registered the trial after the enrolment of the participants started (Identifier nr. NCT03946176). Sixteen volunteers were enrolled according to the following inclusion/exclusion criteria: (i) inclusion criteria were hemodialysis patients, aged between 30 and 65 years, with CKD stage V and having undergone Bicarbonate HD for at least 6 months, body mass index (BMI) between 18.5 and 29.9, omnivorous diet, signature of informed consent; (ii) exclusion criteria were use of antibiotics or probiotics 30 days before the recruitment, chronic gastrointestinal diseases, systemic inflammatory disease, suspected or clinical diagnosis of malignancy, chronic liver disease, corticosteroid therapy or immunosuppressive drugs, psychiatric conditions that reduce compliance with therapeutic protocols. At the enrolment (T0), patients were randomized for the use of the synbiotic NATUREN G (*n* = 7) or placebo (*n* = 6) for 8 weeks. NATUREN G® (Farmalabor SRL) consisted of a mixture of probiotics (*Lactobacillus Casei* LC4P1 3.2 × 10^9^, *Lactobacillus Bulgaricus* SP5 3.2 × 10^9^, and *Bifidobacterium Animalis* BLC1 3.2 × 10^9^), prebiotics (fructoligosaccharides 5 g and inulin 5 g), and natural antioxidants (a mix of quercetin 0.13 g, resveratrol 46 ug, and proanthocyanidins 25 ug). During their last week of either symbiotic or placebo supplementation, all the patients underwent a cycle of three hemodialysis sessions using DVB-PVP cartridge (DVB HD). At T0, after taking the synbiotic/placebo (T7w) and after dialysis with DVB-PVP (T8w), routine blood parameters were evaluated, while blood and fecal samples were collected. At each time point, information about food habits, by means of a food frequency questionnaire, were collected, while Gastrointestinal Symptom Rating Scale and Bristol stool scale questionnaires were administered by the patients to evaluate gastrointestinal symptoms and stool type, respectively. IS and PCS plasma levels were measured pre- and post-hemodialysis at each time point, as reported in Figure 3. Patients were blinded for the use of the synbiotic/placebo, while they were aware of the use of innovative sorbent cartridges. At the end of the innovative hemodialysis cycle, patients were returned to traditional dialysis. The efficacy of innovative hemodialysis was compared with standard bicarbonate hemodialysis (BHD) for each patient.

Primary outcome: change in serum concentration of PCS. Secondary outcomes: change in serum concentration of IS, inflammatory markers. Exploratory outcomes: modulation of gut microbiota composition, change in gastrointestinal (GI) symptoms and stool type, change in serum concentration of trimethylamineoxide. The study was carried out at the ambulatories of the Nephrology and Dialysis Unit of our Department. No incentive was provided to the volunteers. 

RRs for IS and PCS were calculated according to the following equation:

RR = (1 − Cpost-corr/Cpre) × 100 (%)

using corrected Cpost (Cpost-corr) as reported by D. H. Krieter and coworkers [16]. Cpost was corrected for extracellular volume changes based on differences in the patient’s pre- (BWpre) and post-dialysis body weight (BWpost) as the following equation: 

Cpost-corr = Cpost/(1 + ((BWpre − BWpost)/0.2 × BWpost)) (mg/L).

### 4.5. Dialysis Modalities

The dialysis modality before inclusion was BHD for all patients for at least 6 months. BHD was performed with low-flux polysulfone (PS) membranes (F8- HPS Fresenius Medical Care, Bad Homburg, Germany) with a surface area of 1.8 m^2^ and a blood flow of 300 ± 50 mL/min and a dialysate flow of 550 ± 100 mL/min, dialysis time 240 ± 15 min. The composition of the dialysate was sodium 140 mmol/l, potassium 2.0–3.0 mmol/l, bicarbonate 32 mmol/l, calcium 1.5 mmol/l, magnesium 0.5 mmol/l, chloride 111 mmol/l, acetate 3 mmol/l, and glucose 1 g/L. The dialysate was purified by ultrapure filtration before entering the dialyzer. Net fluid removal was set on an individual basis according to the patient’s clinical needs. All dialytic treatments were carried out with a volumetric control machine allowing for a precise rate of fluid removal. The analyses of the dialysis water were performed monthly, ascertaining the absence of bacteria (<100 colony forming units/ml) or bacteriological contaminant products (endotoxin levels <0.025 endotoxin units). The innovative DVB-PVP HD treatment was performed in combination with the dialytic treatment, inserting the sorbent cartridge in series before the dialyzer on the main extracorporeal circuit, to work directly on whole blood, without changing the usual technical settings.

Anticoagulation was performed with sodium eparin. For each patient, the dialysis prescription was kept constant throughout the study (total dialysis time, dialysate flow, dialysate temperature, and dialysate composition), and the blood flow was kept as stable as possible. Clinical monitoring included intradialytic symptoms (symptomatic hypotension, episodes of arrhythmia, and thoracic pain) and hospital admissions for any reason, and withdrawal from the study and their causes.

### 4.6. Blood Analyses

Blood samples were processed for routine analyses, including metabolic parameters, such as glycemia, which were measured using Siemens enzymatic methods (Dimension Vista 1500, Siemens Health Diagnostics, Deerfield, IL). An aliquot of blood for each patient/time point was centrifuged at 2000×g for 10 minutes to obtain plasma samples, which were then stored at −80 °C until use.

### 4.7. LC-MS/MS for Quantification of PCS and IS

IS and PCS were assayed in the experimental solution, before the perfusion, and after 1, 3, and 6 h perfusion in pre- and post-cartridge samples (Figure 1). Pre-dialysis and post-dialysis IS and PCS plasma levels were determined at each time point, as reported in Figure 3 and Figure 4. Total and free plasma levels of IS and PCS were assayed by multiple reaction monitoring (MRM) analysis, using a triple quadrupole mass spectrometer (API4000, AB SCIEX, Carlsbad, CA, USA) equipped with an electrospray ionization source and an on-line connected to high-performance liquid chromatography system (CBM-20A LC, Shimadzu, Kyoto, Japan), as previously described by our group [12]. All samples were run in triplicate.

### 4.8. Statistics

A Mann–Whitney U test was used to analyze differences in IS and PCS levels in the in vitro study and between groups in the in vivo study. Baseline and follow-up parameters and variables of hemodialysis patients were compared between groups by the Wilcoxon signed rank test as appropriate for continuous non-parametric data.

Linear regression analysis determined the correlation between chemical and biochemical variables. Results have been considered statistically significant with *p* < 0.05. STATVIEW was used for all statistics.

## Figures and Tables

**Table 1:** Mean value of the total mass removal after 6 hours passing through the cartridge containing the resin. MB: Mass Balance = (Cfinal-Cinitial) × Total Volume – RR (%): Removal Rate = (Cfinal − Cinitial)/Cinitial × 100. BSA, bovine serum albumin.

Solutes		Total Amount		
		At start	Removed by DVB-PVP	Removed by Cellulose
Indoxyl sulfate MB, mg-(RR%)	Total	75.4	40.9-(54%)	19.1-(25%)
	Free	11.8	8.2-(66%)	1.7-(15%)
p-cresyl sulfate MB, mg-(RR%)	Total	115.7	62.6-(56%)	38.7-(31%)
	Free	14.7	10.4-(70%)	2.3-(15%)
BSA MB, g-(RR%)		126	17.7-(14%)	10.6-(8%)

**Table 2:** Demographic, clinical, and biochemical data of hemodialysis patients at baseline (T0) and at each time point: after 7 weeks of treatment with synbiotic or placebo (T7w), and after the eighth week of treatment with synbiotic or placebo combined with innovative dialysis (T8w). BHD: Bicarbonate Hemodialysis; DVB-PVP HD: DVB-PVP-cartridge hemodialysis; sCr: serum creatinine; CRP: C-reactive protein. * *p* < 0,05 DVB HD vs. BHD; ^§^ *p* < 0.05 DVB HD vs. Baseline (Wilcoxon signed rank test).

Patients Data	Study Population			Controls		
	Baseline (T0)	BHD (T7w)	DVB-PVP HD (T8w)	Baseline (T0)	BHD (T7w)	DVB-PVP HD (T8w)
	Patients treated with Synbiotic (*n* = 6)			Patients treated with Placebo (*n* = 5)		
Age, years	56 ± 7			52 ± 5		
Male, n(%)	5 (83%)			4 (80%)		
SCr, mg/dL	8.5 ± 1.2	8.1 ± 1.5	8.4 ± 1.1	9.4 ± 1.5	8.5 ± 2.4	7.5 ± 2.7^§^
Albumin, g/dL	3.6 ± 0.4	3.7 ± 0.2	3.3 ± 0.4*	3.4 ± 0.4	3.3 ± 0.4	3.0 ± 0.3*^,§^
Azotemia mg/dL	132 ± 44	125 ± 20	114 ± 19	120 ± 33	114 ± 42	100 ± 54
Glycemia, mg/dL	51 ± 19	48 ± 30	79 ± 16*^,§^	72 ± 12	80 ± 20	84 ± 23
CPR mg/L	6.4 ± 5.1	3.1 ± 0.4	5.8 ± 3.9	10 ± 14	10 ± 9	10 ± 7

## Supplementary Materials

The following are available online at https://www.mdpi.com/2072-6651/12/3/170/s1, Table S1: %PB of IS and PCS, and pH values registered in the experimental solution at start condition and at the end of the considered time points. %Δ = (final value – Initial value)/initial value × 100; Figure S1: Plasma concentrations of the free IS and PCS in the two groups (**A** Synbiotic and **B** Placebo) of HD patients; Table S2: Plasma concentrations of the free IS and PCS in the two groups (**A** Synbiotic and **B** Placebo) of HD patients; Table S3: Pre- and post- %PB of IS and PCS, and pH values registered in the plasma of HD patients undergoing DVB-PVP HD beside synbiotic or placebo treatment.
