# Supplementary material for: Efficacy of Divinylbenzenic Resin in Removing Indoxyl Sulfate and P-cresol Sulfate in Hemodialysis Patients: Results from an In Vitro Study and an In Vivo Pilot Trial (xuanro4-Nature 3.2)
Source: Toxins (Basel). 2020 Mar 10;12(3):170. doi: 10.3390/toxins12030170 (PMC7150912; doi:10.3390/toxins12030170)
Supplement: Supplementary file 1 [file toxins-12-00170-s001.pdf]

Supplementary Materials: Efficacy of Divinylbenzenic Resin in Removing Indoxyl Sulfate and P-cresol Sulfate in Hemodialysis Patients: Results from an In Vitro Study and an In Vivo Pilot Trial (xuanro4-Nature 3.2)

Maria Teresa Rocchetti, Carmela Cosola, Ighli di Bari, Stefania Magnani, Vanessa Galleggiante, Letizia Scandiffio, Giuseppe Dalfino, Giuseppe Stefano Netti, Mauro Atti, Roberto Corciulo and Loreto Gesualdo

RESULTS

In Vitro Study

Effect of the Resins on the Variation of IS and PCS Albumin Binding

In Table S1 we reported that the percentage of IS and PCS albumin binding (%PB) increased during the six hours of perfusion through DVB-PVP, reaching, after 6 hours, a peak of 5.7% and 7.8% of the starting value for IS and PCS, respectively. Conversely, six hours of perfusion through Cellulose decreased by 0.4% and 0.9% the IS and PCS percentage of albumin binding, respectively. At the same time, we noticed an increase of 5.1% of the initial pH of the experimental solution after six hours of perfusion through DVB-PVP and a decrease of 0.5% of the initial pH after six hours of perfusion through Cellulose. We found a positive correlation between IS and PCS %PB and pH ($r = 0.78$, $p = 0.01$ and $r = 0.82$, respectively, $p = 0.0006$ for both) in DVB-PVP perfusion.

Table S1. %PB of IS and PCS, and pH values registered in the experimental solution at start condition and at the end of the considered time points. %Δ = (final value – Initial value)/initial value × 100.

Title	At Start	DVB			%Δ	CELLULOSE			% Δ
	(T0)	T1h	T3h	T6h		T1h	T3h	T6h	
%PB IS	84.15	87.3	88.1	89.0	5.7	81.3	82.5	83.8	−0.4
%PB PCS	87.15	87.2	93.2	94.0	7.8	83.5	84.9	86.3	−0.9
pH	5.84 ± 0.02	5.89 ± 0.2	6.12 ± 0.01	6.15 ± 0.01	5.1	5.85 ± 0.04	5.84 ± 0.04	5.83 ± 0.02	−0.5

Clinical Trial

Evaluation of Free IS and PCS Plasma Levels in Group A and B

Free IS and PCS plasma levels followed the same trend of reduction as the total IS and PCS, (Figure S1). In all pre-dialysis plasma samples, the free portion of IS and PCS corresponded to a mean value of 3.6 ± 0.3% of total IS and 2.8 ± 0.2% of total PCS in the group A (symbiotic) and 3.3 ± 0.8% of total IS and 2.4 ± 0.5% of total PCS in the group B (placebo). Instead, in all post-dialysis samples, we observed lower percentages of free IS (1.9 ± 0.2% of total IS) and PCS (1.7 ± 0.4% of total PCS) in the group A and 2.1 ± 0.6% of total IS and 1.4 ± 0.4% of total PCS in the group B suggesting a delay in restoring the balance between the two forms after dialysis. Differences in percentages of the free IS and PCS were not statistically significant between groups (Synbiotic vs placebo, Mann-Whitney u test). Two of the five patients of the group B (placebo) did not complete the third DVB-PVP HD session owing to lowering of blood pressure during the treatment, probably not allowing to observe the trend towards reduction of IS and PCS levels also after the third experimental session (last column, Figure 3B).

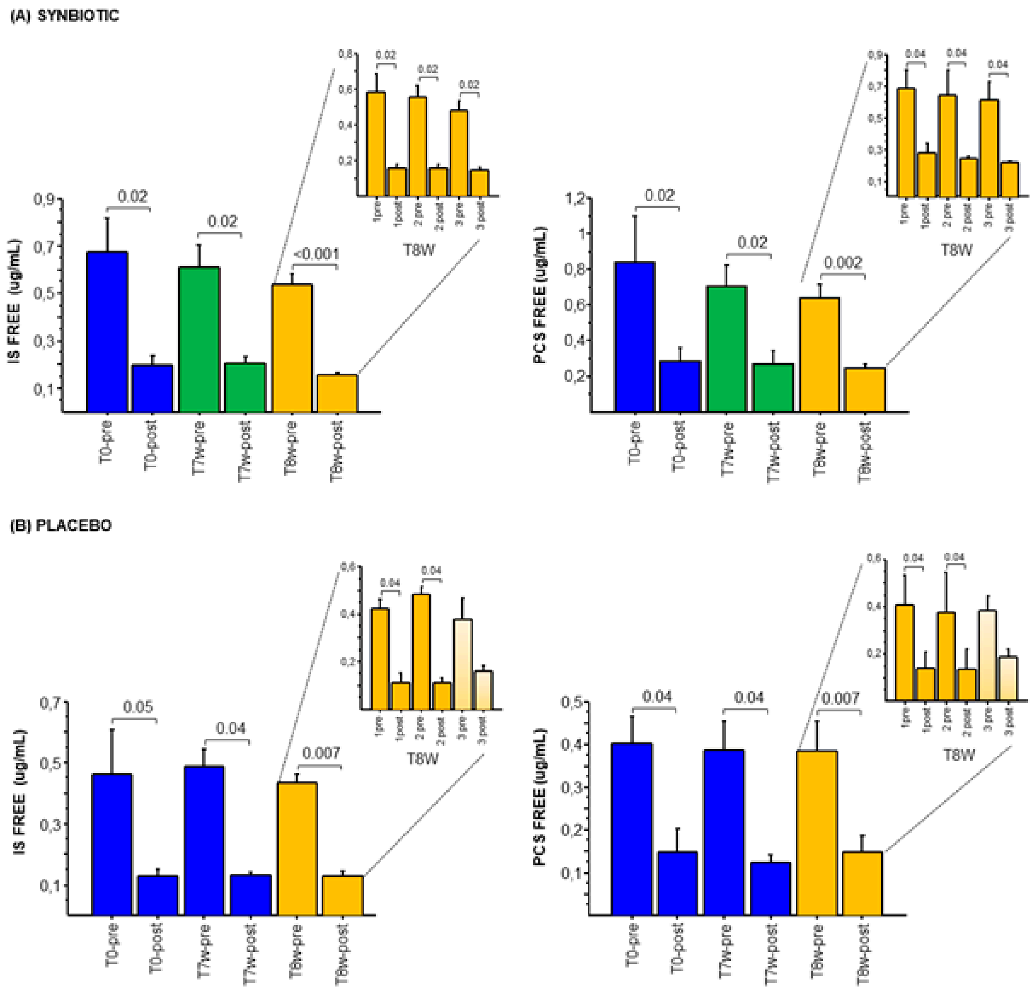

Figure S1. Plasma concentrations of the free IS and PCS in the two groups (**A** Synbiotic and **B** Placebo) of HD patients: (**A**) at baseline (T0-pre/post dialysis); after 7 weeks of synbiotic treatment (T7w-pre/post dialysis); mean values before and after the three dialysis sessions with DVB-cartridge with the synbiotic treatment at week 8 (T8w-pre/post dialysis). At the top right of each chart are reported the values before and after each dialytic session carried out at week 8: first (1-pre/post), second (2- pre/post) and third (3-pre/post) dialysis session. (**B**) at baseline (T0-pre/post dialysis); after 7 weeks of placebo treatment (T7w-pre/post dialysis); mean values before and after the three dialysis sessions with DVB-cartridge with the synbiotic treatment at week 8 (T8w-pre/post dialysis). At the top right of each chart are reported the values before and after each dialytic session carried out at week 8: first (1-pre/post), second (2- pre/post) and third (3-pre/post) dialysis session. Data are expressed as mean ± SE. (Wilcoxon test).

Evaluation of the Reduction Ratio (RRs) for IS and PCS after Each Dialysis Session

The RRs for total and free portions of IS and PCS was evaluated in both study's groups (A and B) as described in Methods paragraph. All value are reported in Table S2. The lack of significance of all the RR% differences it's probably due to the variability of the data in the small study population.

Data of the third DVB-PVP HD session are not very indicative because they are only for three of five patients as described in results' paragraph.

Table S2. Plasma concentrations of the free IS and PCS in the two groups (**A** Synbiotic and **B** Placebo) of HD patients

**Title**		**Indoxyl Sulfate**					**p-Cresyl Sulfate**				
		BHD		DVB-PVP HD			BHD		DVB-PVP HD		
		T0	T7w	T8w1	T8w2	T8w3	T0	T7w	T8w1	T8w2	T8w3
Group A Synbiotic	RR (%) Tot	42 ± 8*	42 ± 6*	41 ± 5*	45 ± 6*	44 ± 8*	37 ± 7*	38 ± 4*	37 ± 5*	41 ± 5*	41 ± 10*
	RR (%) Free	71 ± 2	66 ± 2	71 ± 3	72 ± 1	70 ± 1	62 ± 3	63 ± 3	72 ± 3	70 ± 3	70 ± 2
Group B Placebo	RR (%) Tot	37 ± 10*	38 ± 11*	54 ± 8*	48 ± 9	45 ± 7	38 ± 8*	39 ± 11	49 ± 8	46 ± 11	31 ± 9
	RR (%) Free	68 ± 8	71 ± 4	70 ± 10	74 ± 3	50 ± 3	64 ± 8	67 ± 2	65 ± 5	63 ± 9	46 ± 2

*: Differences between total and the corresponding free RRs were statistically significant with $p < 0,05$ (Mann-Whytney U test).

Evaluation of IS and PCS Protein Binding (%PB) during the DVB-PVP HD Treatment

As well as in the in vitro study, we evaluated the variation of IS and PCS protein binding (%PB) during the DVB-PVP HD treatment, either in conjunction (group A) or not (group B) with the synbiotic. We observed an increased percentage of IS and PCS protein binding in post-dialysis samples along with an increase in pH (Table S3). Indeed, we found a positive correlation between the pH of plasma and IS and PCS %PB, independently from the simultaneous administration of the synbiotic ($r = 0.61$, $p < 0.0001$ for IS; $r = 0.5$, $p < 0.0001$ for PCS, respectively), supporting the in vitro data.

Table S3. Pre- and post- %PB of IS and PCS, and pH values registered in the plasma of HD patients undergoing DVB-PVP HD beside synbiotic or placebo treatment. * $p < 0,05$ DVB-PVP HD post *vs* DVB-PVP HD pre.

	**Study Population Group A (Synbiotic)**					
	DVB-PVP HD T8w					
	1st session - T8w 1		2nd session - T8w 2		3th session - T8w 3	
	pre	post	pre	post	pre	post
%PB IS	96.1 ± 2	98.3 ± 1	96.4 ± 1	98.1 ± 1	96.1 ± 1	98.0 ± 1
%PB PCS	96.7 ± 2	98.7 ± 1	96.9 ± 1	98.5 ± 1	96.7 ± 0.3	98.1 ± 1
pH	7.37 ± 0.06	7.51 ± 0.08*	7.41 ± 0.05	7.50 ± 0.06*	7.39 ± 0.04	7.52 ± 0.04*
	**Controls Group B (Placebo)**					
%PB IS	96.8 ± 3	97.0 ± 3	95.6 ± 3	97.4 ± 2	97.6 ± 1	98.1 ± 1
%PB PCS	98.0 ± 1	98.7 ± 0.5	97.0 ± 1	98.0 ± 1	96.3 ± 0.2	98.6 ± 1
pH	7.39 ± 0.1	7.46 ± 0.06*	7.32 ± 0.08	7.47 ± 0.05*	7.35 ± 0.08	7.46 ± 0.05
